# Two-Signal Set and Adaptive Spectral Decomposition Algorithm for Estimating the Phase Velocity of Dispersive Lamb Wave Mode

**DOI:** 10.3390/s26072190

**Published:** 2026-04-01

**Authors:** Lina Draudvilienė, Asta Meškuotienė, Aušra Gadeikytė, Paulius Lapienis

**Affiliations:** 1 Ultrasound Research Institute, Kaunas University of Technology, K. Baršausko St. 59, LT-51423 Kaunas, Lithuania; plapienis@gmail.com; 2 Metrology Institute, Kaunas University of Technology, Studentu Str. 50, LT-51368 Kaunas, Lithuania; asta.meskuotiene@ktu.lt; 3 Department of Applied Informatics, Kaunas University of Technology, Studentu Str. 50, LT-51368 Kaunas, Lithuania; ausra.gadeikyte@ktu.lt

**Keywords:** ultrasonic Lamb waves, phase velocity dispersion, frequency, zero-crossing technique, spectrum decomposition technique

## Abstract

This study introduces an automated computational tool to evaluate the phase velocity of the highly dispersive *A*_0_ mode using only two signals measured along the wave propagation path. The algorithm combines the zero-crossing technique with automated spectral decomposition, utilizing a bank of bandpass filters with adaptive bandwidths. Validated through theoretical and experimental analysis of an aluminium plate near 300 kHz, the results demonstrate that using a two-signal set and variable filter widths significantly improves accuracy and extends the measurable frequency range of the dispersion curve. Experimental results demonstrate that by applying various filter widths, the phase velocity dispersion curve segment can be reconstructed over a frequency range exceeding 65% of the signal’s spectral width at the −40 dB level. The reconstruction yielded an average relative error of 0.8% ± 1.2%, while the best-case scenario showed an error of just 0.3% ± 0.4%. Implementing automated filter parameter selection on a signal pair offers a time-efficient alternative to traditional spatial scanning, significantly simplifying data collection while reducing labour and time requirements.

## 1. Introduction

The use of Lamb waves in various industry areas for the accurate detection of defects is a technology that has been known for quite some time. Non-stationary Lamb wave signals are highly sensitive to changes in material properties; therefore, such signals are used for the determination of the object condition [1,2]. These waves can also be effectively utilized to monitor the stress state of plates and structural components, as dispersion characteristics are sensitive to stress-induced changes in material stiffness [3]. The phase velocity of these waves is a key parameter for assessing structural changes and is widely utilized in non-destructive testing (NDT). Accurate determination of phase velocity in both defective and non-defective regions is a prerequisite for precisely localizing and characterizing defects. However, due to the unusual nature of Lamb waves, analyzing the received signals is a challenging task. Phenomena such as dispersion, an infinite number of modes, mode convergence and interlacing, reflection, refraction, mode conversion, edge-induced splitting, and scattering [2,4,5] distort the signal waveform or mask the target signal, making it difficult to extract the necessary information. These effects are further influenced by specimen geometry, material properties, and environmental or operating conditions [1]. Consequently, extracting information from Lamb wave signals requires addressing two primary challenges. The first is to identify the useful signal in the obtained signal set. The second is to apply appropriate signal processing methods (SPMs) to obtain the necessary information. Given that this study focuses on the development of an SPM for phase velocity determination, the associated difficulties and the parameters that must be evaluated to properly determine the phase velocity of Lamb waves are discussed.

The main unusual feature of dispersive Lamb waves is that different frequency components of the signal propagate at varying velocities [4]. Consequently, the phase velocity of a Lamb wave mode is a function of both the object’s thickness and the frequency. This relationship is represented by the phase velocity dispersion curve [5,6]. Various analytical and/or semi-analytical methods have been developed and used to calculate dispersion curves [6,7]. Knowing the elastic and geometric properties of an object, it is possible to calculate dispersion curves with sufficient accuracy using any of these methods and theoretically determine the phase velocity in the object under analysis. These theoretical methods provide a robust basis for choosing excitation frequencies and assessing the accuracy of developed signal processing techniques [8]. However, theoretical dispersion curves represent idealized models and do not account for experimental signal parameters such as Time-of-Flight (ToF) or time–frequency (t-f) characteristics. Experimental measurements are essential to obtain the comprehensive and reliable data needed for accurate wave propagation analysis and defect detection. Extracting this information from physical structures requires signal processing methods capable of handling the inherent complexity of Lamb waves. Standard signal processing methods (SPMs), however, are often inadequate for determining phase velocity due to the dispersive nature of Lamb waves. While numerous SPMs have been established—including two-dimensional Fast Fourier Transform (2D-FFT) and its variations [9,10], matrix pencil method [11], zero-crossing or tracking the peaks of the pulse techniques [12,13], multi-signal classification (MUSIC) [14], etc. To obtain a segment of the phase velocity dispersion curve, most existing techniques require signal recording at multiple points along the wave propagation path, and in other cases, they are not reliable [15]. Consequently, object scanning is time-consuming and demands human and energy resources. To address these limitations, new methods are being developed to simplify the data acquisition process by providing a time- and cost-efficient alternative to traditional spatial scanning.

Recently, new SPMs have been developed that enable phase velocity estimation using only a set of two signals [16,17]. Since they are still only in the research and testing stage, the developed methods have many limitations, are not precise enough, or lack a clear and precise methodology. Thus, some methods are used for the phase velocity evaluation using only two signals. Notably, Zeng [16] proposed the short-time chirp-Fourier transform (STCFT) method. While a specialized chirp signal was proposed for Lamb wave generation, this approach complicates the isolation of individual modes at varying propagation distances. Furthermore, several critical parameters for phase velocity calculation are omitted, such as the rationale for the distance between signal acquisition points and the specific signal characteristics that dictate point selection. The authors’ experimental errors obtained in certain frequency ranges reach up to 10%, which indicates that the method is not reliable [16]. Another proposed approach combines the Hilbert transform with cross-correlation to estimate the phase velocities of the *A*_0_ and *S*_0_ modes [17]. However, the reliance on cross-correlation makes this method highly sensitive to waveform distortion, particularly over long propagation distances. As non-stationary Lamb waves travel, they undergo dispersion, which causes the signal amplitude to decrease and the pulse to elongate [18]. Furthermore, the study [17] does not provide a formal quantification of the method’s reliability.

We previously developed a hybrid approach combining the zero-crossing method with spectral decomposition to estimate the phase velocities of dispersive Lamb wave modes using a signal pair [19]. This combined technique successfully reconstructed the phase velocity curve of the dispersive asymmetric mode over a broad frequency range, achieving a mean relative error of approximately 1% [19]. However, subsequent analysis has revealed that the selection of an appropriate signal pair is the critical factor for accurate phase velocity segment reconstruction. While utilizing a pre-acquired signal array makes it straightforward to select suitable signal pairs for phase velocity reconstruction, the primary challenge lies in identifying these pairs without the need for an exhaustive scan of the object. To overcome this limitation, the algorithm was modified, and additional studies were conducted.

This work aims to develop and present a signal processing method that allows the reconstruction of a segment of the phase velocity dispersion curve using a set of two signals measured along the Lamb wave propagation path; to confirm the capabilities of the proposed method using asymmetric *A*_0_ mode signals generated in the high-dispersion region; and to perform an analysis of the reliability errors and uncertainties of the method.

The paper is organized as follows: an algorithm for the phase velocity evaluation is presented in Section 2. The theoretical study of the proposed method is presented in Section 3. Experimental verification is described in Section 4. Evaluation of method reliability characteristics is discussed in Section 5. Conclusions of the study are presented in Section 6.

## 2. Algorithm for the Phase Velocity Evaluation

### 2.1. The Filtering of the Signals

To extract the required information from only the two measured signals, filtering using a chosen bandpass filter package was proposed [19]. An algorithm based on a combination of zero-crossing and spectrum decomposition techniques was applied. The proposed algorithm consists of two main parts. A brief description of the early proposed algorithm:

1. The filtering is conducted using the spectrum decomposition technique:
  - Two signals *u*_1_ (*t*), *u*_2_ (*t*) are fixed.
  - The frequency spectra for both acquired signals are computed using the Fourier transform.
  - The filtering is performed using Gaussian filters [20]. Based on the obtained frequency spectra, the frequency ranges in which filtering is performed are determined, the central frequencies of the filters are selected, and the filter bandwidth is determined. The number of filtering operations is calculated according to the expression presented by He [20]:
    (1) $$b\geq 1+\frac{f_{H}-f_{L}}{B}.$$
    where *f_L_* and *f_H_* define the frequency ranges in which the central frequencies of the filters are varied; *B* is the filter bandwidth.
  - The filtered signals are restored using the Inverse Fourier transform.
2. The zero-crossing technique is employed to calculate a point of the phase velocity dispersion curve:
  - The threshold level *L* is determined.
  - The phase velocity value (*c_ph_*) is calculated using the time instants at which both signals cross the zero-amplitude line $t_{1}(x_{1})$ and $t_{1}(x_{2})$:
    (2) $$c_{ph}=\frac{x_{2}-x_{1}}{t_{2}\left(x_{2}\right)-t_{1}\left(x_{1}\right)},$$
  - The equivalent frequency value, which corresponds to the calculated duration of half-period of the second signal, is estimated using the time instants at which the signal crosses the zero-amplitude line $t_{1}(x_{2})$, $t_{2}(x_{2})$ by:
    (3) $$T_{0.5}\left(x_{2}\right)=t_{2}\left(x_{2}\right)-t_{1}\left(x_{2}\right)$$
  - The frequency value ($f_{0.5,k}$) is determined:
    (4) $$f_{0.5}=\frac{1}{{2T}_{0.5(x_{2})}}$$
  - The point of the phase velocity dispersion curve is determined, which is described by creating a set of pairs of frequency $f_{0.5}$ and determined phase $c_{ph,k}$ velocity values:
    (5) $$\left\{{f_{0.5},c}_{ph}\right\}.$$

This algorithm is repeated for each of the selected filters. Accordingly, the frequency range of the reconstructed phase velocity dispersion curve segment directly correlates with the number of filters employed. By utilizing the proposed signal filtering algorithm, a sufficiently wide frequency range can be reconstructed from only a single pair of signals. However, several factors complicate the application of this algorithm. The following example illustrates these challenges.

**Figure 1:**
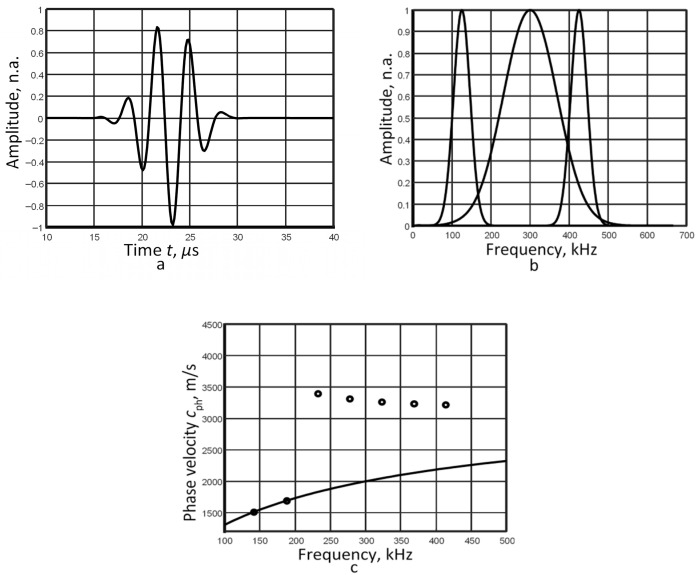
Signal at a distance of 48 mm (**a**), the frequency spectrum and the band pass filter with central frequencies of 125 kHz and 425 kHz (**b**), and the reproduced phase velocity dispersion curve (**c**).

For this study, a 2 mm thick aluminium plate with a density *ρ* of 2710 kg/m^3^, Young’s modulus *E* of 71.7 GPa, and Poisson’s ratio *ν* of 0.33 is used. The excitation signal *u*_0_(*t*), a 3-period, 300 kHz harmonic burst with a Gaussian envelope, is used as the incident signal. We focused on the *A*_0_ Lamb wave mode, which is characterized by high dispersion in the specified frequency range. The analysis utilizes a pair of signals captured at distances of 30 mm and 48 mm. The waveform and frequency spectrum of the signal at 48 mm are presented in Figure 1a and Figure 1b, respectively.

Based on the algorithm, the filter bandwidth and central frequencies are selected. According to the obtained frequency spectrum (Figure 1b), the bandwidth of the signal at the −40 dB level is in the range of 100–500 kHz. The filter bandwidth Δ*B* is set to 50 kHz. Seven filtering operations are determined (Equation (1)), and the following central frequencies are chosen: 125 kHz, 175 kHz, 225 kHz, 275 kHz, 325 kHz, 375 kHz, and 425 kHz. The zero-crossing method is applied in each filtering case. The resulting set of phase velocity and frequency (Equation (5)) represents a single point on the phase velocity dispersion curve for each filtering operation. The calculated phase velocity results are compared with the dispersion curve obtained via the SAFE method, as shown in Figure 1c.

The results demonstrate that the constant filter bandwidth restricts the reconstruction of the phase velocity dispersion curve; consequently, only two points (at 125 kHz and 175 kHz) are accurately resolved (Figure 1c). Thus, this issue suggests a modification of the algorithm, where the application of variable-width filters could be an effective solution to evaluate the phase velocity of the dispersive mode.

### 2.2. Modified Algorithm

**Figure 2:**
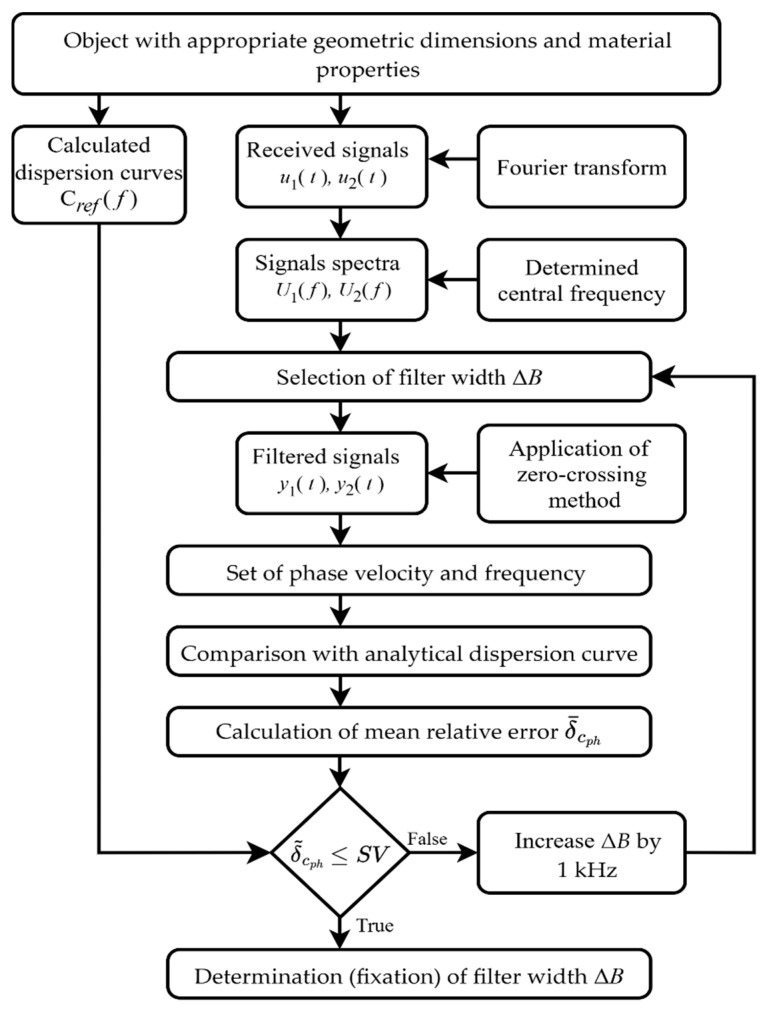
Algorithm for calculating filter bandwidth according to the selected central frequency.

To estimate the phase velocity dispersion using a determined set of two signals, a suitable width of the filter bandwidth is proposed to calculate for each selected central frequency. The algorithm for determining the filter bandwidth for selected central frequencies is shown in Figure 2 and explained in detail below.

- Once the two signals are selected, the maximum distance ∆l between them is determined based on the object geometry, material properties, and frequency range of the study. The performed study shows that this distance can be determined from the dispersion curves of the phase and group velocities at the central frequency accordingly [13]:
  (6) $$∆l\leq \frac{1}{f}\cdot \frac{c_{gr}(f)\cdot c_{ph}(f)}{\left|c_{gr}(f)-c_{ph}(f)\right|}$$
  where $c_{gr}(f)$ is the group velocity at the central frequency, and $c_{ph}(f)$ is the phase velocity at the central frequency.
- The central frequencies are determined. Depending on how wide the phase velocity dispersion curve should be reproduced and how many points are needed to determine its nature, the number of central frequencies is selected.
- The width of the filter bandwidth is set. According to the presented algorithm (Figure 2), the width of the filter bandwidth is determined for each selected central frequency using the same set of two signals.
- The filtered signals are restored using the Inverse Fourier transform.
- The zero-crossing technique is applied to obtain a set of phase velocities and frequencies.
- Each time, the result obtained using a different width of the filter bandwidth at the selected central frequency is compared with the calculated analytical dispersion curve. This comparison of the obtained values shows whether the phase velocity is calculated correctly [20]. If the result deviates from the analytical value, the algorithm iterates through the sequence using a modified bandwidth until a match is achieved.
- The calculated average relative error indicates whether the selected filter width is appropriate. Therefore, it is essential to first establish a maximum permissible error and then select the filter according to that limit. In this theoretical context, a maximum relative error of 1% is used. After obtaining a set of phase velocity and frequency values that meet the criterion SV, the calculation of the remaining values is performed according to the described algorithm.
- The algorithm is repeated for each specified central frequency.

## 3. Theoretical Study

The same aluminium plate, with parameters detailed in Section 2.1, is employed for the proposed algorithm study. Three sets of signals, corresponding to distances of 25 mm and 43 mm, 48 mm and 66 mm, and 95 mm and 113 mm, are selected for analysis. The frequency spectra of these signals match those shown in Figure 1b. Seven central frequencies (150 kHz, 200 kHz, 250 kHz, 300 kHz, 350 kHz, 400 kHz, and 450 kHz) are considered. Based on the algorithm (Figure 2), the appropriate filter bandwidth was determined for each frequency; these values are presented in Table 1.

Our previous work [13] describes the application of the zero-crossing method and the selection of an appropriate threshold level *L* for reconstructing phase velocity curves from a signal pair. This approach introduces a flowchart for determining the optimal threshold. This threshold is used to identify the correct Time-of-Flight (ToF) values for both signals based on zero-crossing points, subsequently enabling the calculation of phase velocity values (*c_ph_*). The brief description of an algorithm is presented below.

The appropriate threshold level *L* is determined based on the proposed flowchart [13].

Time instants at which both signals cross the zero-amplitude line $t_{1}(x_{1})$, $t_{2}(x_{1})$, …, $t_{N}\left(x_{1}\right)$ and $t_{1}(x_{2})$, $t_{2}(x_{2})$,…, $t_{N}(x_{2})$ are measured. The phase velocity values are calculated according:

(7) $$c_{ph,k}=\frac{x_{2}-x_{1}}{t_{k}\left(x_{2}\right)-t_{k}\left(x_{1}\right)},$$

where *k* = 1, 2 … *N*; *k* is the number of zero-crossing instants in the signals; *N* is the total number of measured zero-crossing instants in the signals.

- The equivalent frequencies are estimated based on the calculated durations of the selected half-periods of the second signal. The duration of the second signal, with selected half-periods $T_{0.5,1}(x_{2})$, $T_{0.5,2}(x_{2})$, …, $T_{0.5,N-1}(x_{2})$, is calculated:
  (8) $$T_{0.5,k}\left(x_{2}\right)=t_{k+1}\left(x_{2}\right)-t_{k}\left(x_{2}\right)$$
  The frequency values ($f_{0.5,k}$) for the dispersion curve reconstruction are determined:
  (9) $$f_{0.5,k}=\frac{1}{{2T}_{0.5,k(x_{2})}}$$
- The phase velocity dispersion curve segment is obtained, which is described by creating sets of pairs of frequencies $f_{0.5,k}$ and determining the phase $c_{ph,k}$ velocities:
  (10) $$\left\{{f_{0.5,k},c}_{ph,k}\right\}.$$

Studies using both theoretical and experimental signals validated the method’s efficacy in reconstructing phase-velocity dispersion curves [13]. Since the phase-velocity dispersion curve segment can be reconstructed using both tools with the same two measured signals, an additional study was conducted to determine which is more effective. Thus, by applying the algorithm presented in [13], the threshold level is determined for each selected set of signals. The results are presented in Table 2. The results obtained by using filtering are presented in Table 3.

After applying both tools, the segments of the phase velocity dispersion curves are restored, their widths are calculated, and the results are presented in Table 2. To evaluate the reliability of the tools, the maximum deviation from the mean velocity error (${∆}_{c_{phmax}}$) and mean relative error (${\bar{\delta }}_{c_{ph}}$) is calculated in each case. The mean relative error is calculated accordingly:

(11) $${\bar{\delta }}_{c_{ph}}=100\%\cdot \frac{1}{N}\sum_{n=1}^{N}\frac{\left|c_{phn}-c_{ph}^{SAFE}\right|}{c_{ph}^{SAFE}},$$

where *c_ph_* represents the phase velocity values calculated by the presented algorithms, and $c_{ph}^{SAFE}$ is the value of the phase velocity calculated by the SAFE method.

**Figure 3:**
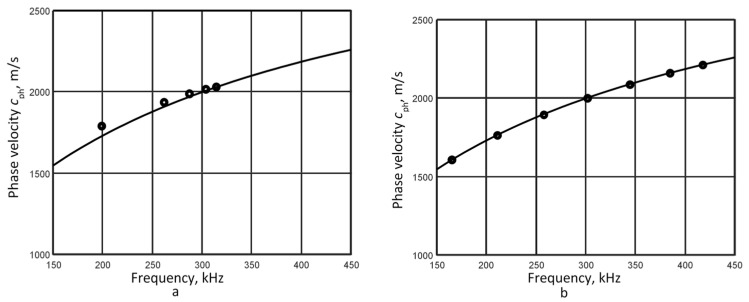
Reconstructed *A*_0_ mode phase velocity dispersion curves from signals at 48 mm and 66 mm: (**a**) without filtering and (**b**) with filtering.

The reconstructed segments of the *A*_0_ mode phase velocity dispersion curve are shown in Figure 3. The results without filtering are in Figure 3a, while the results with filtering are in Figure 3b.

The calculated average relative error, as presented in Table 3, demonstrates that the proposed use of filters with varying bandwidths is a viable solution for evaluating phase velocity dispersion across a wider frequency range with reduced error. Using filtering on signals closer to the excitation source (first case), the phase velocity dispersion segment is recovered over a frequency range more than four times wider, with a mean relative error of 0.1%. It can be explained that the different frequency components of the signal concentrate around the central frequency and propagate at similar velocities at a short distance from the excitation source. Thus, the phase velocity dispersion curve is reconstructed in a very narrow bandwidth around the central frequency of 300 kHz. Since the complex spectrum module (Figure 1b) reflects the amplitude of both higher- and lower-frequency components, higher-amplitude frequency components are filtered depending on the filter width. This increases the sensitivity of the signal processing technique to low-amplitude frequency components.

The comparative analysis demonstrates that filtering signal spectra with varying bandwidths is an effective approach for characterizing the phase velocity dispersion of Lamb waves. The following section presents the experimental validation of this conclusion.

## 4. Experimental Verification

**Figure 4:**
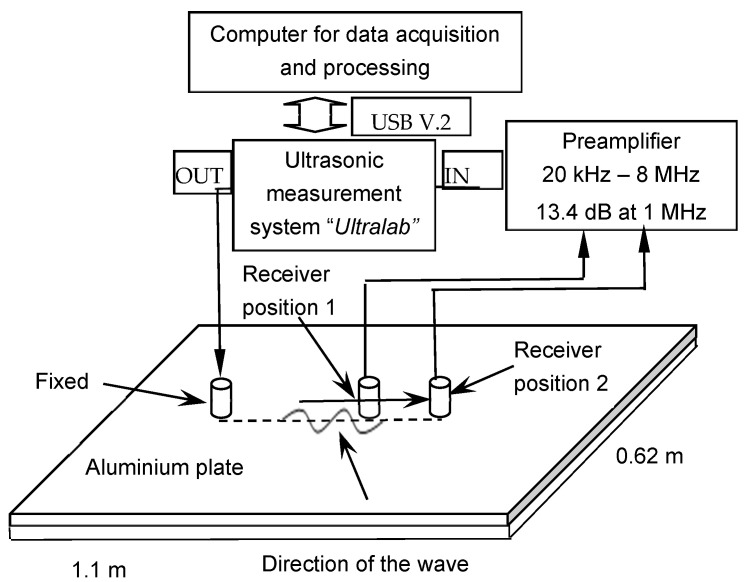
Experimental measurement structural schema.

The experimentally obtained Lamb wave signals, which propagated in an isotropic aluminium plate with material properties presented in Section 2.1, dimensions of (1.1 × 0.62) m^2^, and a thickness of 2 mm, were used for the study. A structural schema of an experimental measurement setup for generating and receiving Lamb wave signals is presented in Figure 4.

The experimental study utilized wideband, contact-type ultrasonic transducers with a resonant frequency of 180 kHz and a bandwidth ranging from 40 kHz to 640 kHz at the −10 dB level [21]. The transmitter was excited by a three-period Gaussian-enveloped pulse, generated and recorded via the ‘Ultralab’ ultrasonic measurement system, which integrates a voltage generator, a low-noise amplifier, and an analog-to-digital converter. A fixed transmitter on the surface of an aluminium plate excited both *A*_0_ and *S*_0_ modes of Lamb waves, and the receiver recorded the signals at selected distances. In the 300 kHz frequency range, the phase velocities of the *A*_0_ and *S*_0_ modes differ, with values of 2000 m/s and 5300 m/s, respectively. This velocity difference facilitated the separation of the signals in the time domain. Thus, five sets of signal pairs at varying Lamb wave *A*_0_ mode propagation distances were selected for the study. In this case, the optimal distances for phase velocity estimation ranged from 15 to 18 mm, corresponding to 75–90% of the maximum calculated distance ∆*l*. Consequently, it is recommended to select a distance between the two signals within the 75–90% range of the maximum calculated value ∆*l*.

To enhance the signal-to-noise ratio (SNR), seven measurements per case were averaged. Signal averaging, a standard technique in ultrasonic testing, suppresses uncorrelated noise [22]. The ultrasonic signal is coherent across acquisitions, while random noise fluctuates, so averaging more waveforms considerably improves signal clarity. Doubling the number of averaged waveforms increases SNR by about 3 dB.

**Figure 5:**
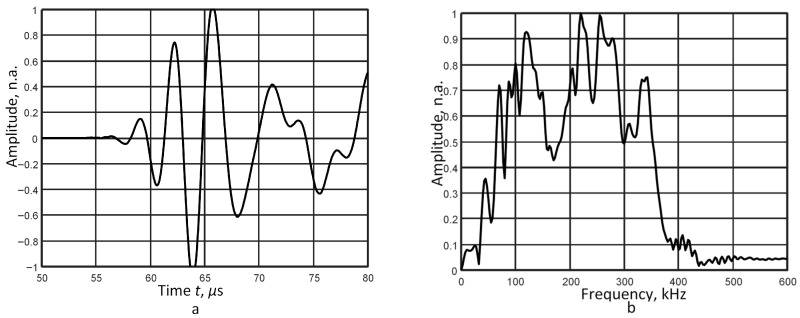
The waveforms of the *A*_0_ mode signal acquired at a distance of 165 mm from the transmitter (**a**) and its frequency response (**b**).

The waveform of the A_0_ mode, acquired at a distance of 165 mm from the transmitter, and its corresponding frequency spectrum are presented in Figure 5a and Figure 5b, respectively. Six central frequencies, 100 kHz, 150 kHz, 200 kHz, 250 kHz, 300 kHz, and 350 kHz, were selected for the study (Table 4).

**Figure 6:**
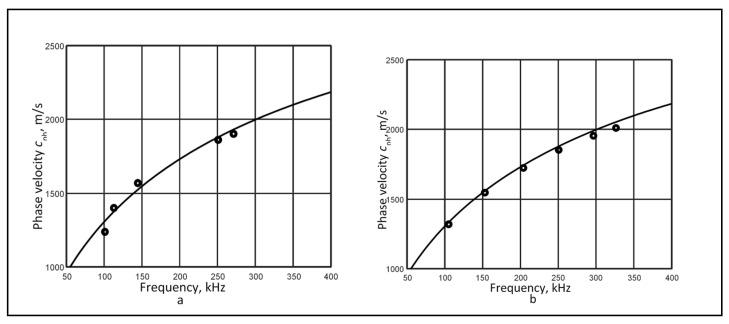
Reconstructed phase velocity dispersion curves of the *A*_0_ mode using a selected pair of two signals at (70–85) mm, without (**a**) and with (**b**) filtering.

The metrological parameters of the reconstructed dispersion curves, obtained from experimental signals, were determined as described in Section 3 and are presented in Table 5. For a specific example, the reconstructed curve segments using a selected pair of two signals at (70–85) mm are shown without filtering in Figure 6a, and with filtering in Figure 6b.

The results in Table 6 demonstrate that filtering the signal spectrum is an effective method for reconstructing phase velocity dispersion curves with greater accuracy and over a wider frequency range. In the first case, filtering expanded the reconstructed frequency range from 155 kHz (114–269 kHz) to 218 kHz (105–325 kHz). This improvement was accompanied by a significant reduction in the mean relative error, from 3.28% without filtering to less than 1.5% with filtering. Other cases also show that filtering with filters of different widths yields significantly better results than without filtering. The error in the average reconstructed frequency ranges decreased from 2.2% to 0.8%. This suggests that filtering is a good solution for obtaining broader and more accurate information from signals that are not yet significantly affected by dispersion, a factor that often complicates data interpretation.

Applying this proposed approach, the evaluation of structural integrity at various stages, such as identifying manufacturing defects in newly produced components or verifying that no damage occurred during transportation before operation, can be used. The essence of this method is to first determine the phase velocity of the Lamb wave propagating in the object in areas free of joints, rivets, defects, or delaminations. Thus, it is necessary to establish the theoretical velocity so that the actual velocity, obtained from real signals in the structure, can be compared against it. Once the actual phase velocity of the Lamb wave in the object is known, other methods can be applied to further verify the integrity of the structure.

## 5. Evaluation of Method Reliability Characteristics

To determine the uncertainty of Lamb wave velocity, potential sources were identified and classified into three categories: methodology implementation, measuring instruments, and specimen properties [8]. To establish the uncertainty budget presented in Table 7, individual uncertainty components were first identified and assigned specific probability distributions, such as a Gaussian distribution for model-related errors and a rectangular distribution for measurement distance. The combined standard uncertainty was subsequently obtained by summing the contributions from all individual sources. Finally, the expanded uncertainty *U* was calculated at a 95% confidence level using a coverage factor of *k* = 2.

Signal noise and distortion, inherent in the dispersion curve recovery process, introduce uncertainty into the final velocity estimation. We will assume that any effects from frequency fluctuations are accounted for within the calculated standard deviation (Table 6).

To evaluate the model’s influence on combined uncertainty, the average standard uncertainty is calculated using simulated signals, as shown in Equation (12):

(12) $$\sigma_{{∆}_{mod}}=\sqrt{\frac{\sum_{n=1}^{N}{\left(\left({∆}_{mod,n}\right)-{\bar{∆}}_{mod}\right)}^{2}}{\left(N-1\right)}},$$

where *N* represents the number of points in a segment of the mathematical reconstructed dispersion curve; *n*th is the point of the segment; ${∆}_{mod,n}$ are the errors of velocities of the reconstructed dispersion curve from the simulated signals; and ${\bar{∆}}_{mod}$ is the average of errors of velocities. The standard uncertainty of reconstructing the real dispersion curve was calculated in the same way, using experimental signals.

The maximum deviation from the mean velocity error ${∆}_{c_{phmax}}$ average is 21.9 m/s in the reconstructed frequency range (101–307) kHz without filtering, and 7.8 m/s in the range (104–341) kHz with filtering. We use this value to estimate the standard uncertainty caused by Lamb wave frequency fluctuations. This value helps assess how the transducer’s operating frequency band affects the constructible frequency range of the dispersion curve. We calculate this component using a Gaussian distribution at a 99% confidence level. This component is calculated as follows: $\sigma \left({∆}_{c_{phmax}}\right)=\frac{{∆}_{c_{phmax}}}{3}$.

Assuming the distance between two signals is determined by the known scanner step of 0.1 mm (Δ*l*), we can calculate the standard uncertainty using a rectangular distribution $\sigma (l)=\pm \frac{∆l}{\sqrt{3}}$. The transducer’s pulse, which excites waves over a specific frequency band, has a duration t defined by the span between the start of the first signal and the end of the second. The influence factor of t is given in Table 7. It is calculated as the partial derivative of the measurement function with respect to the input parameter x_i_, which is equal to 1/t.

Other potential sources of error, such as bandwidth selection and dispersion effects in the filtered signal, are part of the uncertainty budget. The filter width Δ*B* is selected through an iterative process in the modified algorithm. The algorithm adjusts Δ*B* until the mean relative error ${\bar{\delta }}_{c_{ph}}$ meets the set threshold SV (1% in this study). Thus, any error from bandwidth selection is included in the “Mathematical Model” and “Velocity” uncertainty components.

While filtering narrows the frequency band, the *A*_0_ mode remains dispersive within that window. Consequently, different frequency components continue to propagate at slightly different velocities even after filtering. In the uncertainty budget, this effect is captured by the “Fluctuations of the Lamb wave’s frequency” component, which accounts for almost half of the total uncertainty. Therefore, the impact of dispersion within the processed signals is fully reflected in the final expanded uncertainty of ±1.2%.

As such, the material properties were standardized at a reference temperature of 20 °C ± 1 °C. Factors like temperature variations and the specimen’s mechanical and geometric parameters—including density *ρ*, Young’s modulus *E*, Poisson’s ratio *ν*, and plate thickness *d*—can introduce measurement errors by changing the material’s properties [23]. However, this effect is typically insignificant during short laboratory measurements conducted under controlled conditions. Therefore, it’s assumed that these uncertainties are negligible in a controlled laboratory environment. The experiments were performed under controlled normal operating conditions (20 ± 2) °C.

The results presented in Table 7 show that the combined uncertainty is influenced by all four sources identified in the table. The uncertainty budget reveals that the primary contributor to the total measurement uncertainty is the fluctuations of the Lamb wave’s frequency (about 48%), accounting for nearly half of the combined uncertainty. The velocity from the dispersion curve and the distance between two points are also significant contributors, making up 32% and 14%, respectively. The mathematical model has the smallest influence on the overall uncertainty (about 6%). The expanded uncertainty is presented in Table 7 for the mean relative error value, with the dispersion curve reconstructed in the 104–341 kHz interval. In the best-case scenario, the reconstructed curve had an error of 0.3% ± 0.4%.

## 6. Conclusions

A tool to evaluate the phase velocity of the Lamb wave dispersive mode using a set of two signals measured along the wave propagation path is presented in the work. It was proposed to use an integrated zero-crossing and spectrum decomposition technique with an algorithm that allows different filter widths to be set. The theoretical and experimental investigations were performed using asymmetric *A*_0_ mode signals propagating in an aluminium plate at a frequency range of 300 kHz, where this mode exhibits a high dispersive nature. Three sets of signals at different Lamb wave propagation distances were selected for the theoretical study. The calculated average relative error demonstrated that the proposed use of filters with varying bandwidths is a viable solution for evaluating phase velocity dispersion across a wider frequency range, with the obtained error no more than 0.13%. The experimental study was performed using five sets of two signals at different distances. The results of the experiment showed that by using filters of different widths, a segment of the phase velocity dispersion curve can be reconstructed over a sufficiently wide frequency range with an average relative error of less than 1.5%.

This study established an uncertainty budget for Lamb wave A_0_ mode velocity measurements, quantifying the contributions of method implementation, measuring instruments, and specimen parameters. Both simulated and experimental signals were analysed to accurately assess the impact of the reconstruction model and real-world noise. The overall measurement of Lamb wave velocity for the *A*_0_ mode yielded a combined standard uncertainty of 0.6%. This resulted in an expanded uncertainty of ±1.2% at a 95% confidence level (*k* = 2), calculated relative to a phase velocity of 1999 m/s at 300 kHz. In the most favourable scenario, the reconstructed dispersion curve achieved a relative error of 0.3% ± 0.4%. The results proved that accuracy and the constructible frequency range of the dispersion curve are significantly improved by using two-signal sets and applying filters of different widths. The proposed method, nonetheless, has certain limitations. A primary constraint is the requirement for prior knowledge of the object’s elastic properties and geometry to calculate the phase and group velocity dispersion curves. Furthermore, the current performance of this approach has not been evaluated at excitation frequencies where higher-order modes emerge. Nevertheless, the method remains applicable provided the signals are temporally separated and do not overlap in the time domain. Investigating these complex conditions remains a subject for future research.

Summarizing the results obtained, it can be stated that the application of an algorithm that allows different filter widths to be set for the analysis of two set signals is a useful tool, as it avoids object scanning, which simplifies the acquisition of information and saves time.

## Figures and Tables

**Table 1:** Calculated suitable filter bandwidths for the selected central frequency.

Distance Between the Signals (*x*_1_–*x*_2_), mm	Central Frequencies of the Filter, kHz						
	150	200	250	300	350	400	450
	Bandwidth of the Filter Δ*B*, kHz						
25–43	50	50	56	57	66	68	73
48–66	50	50	58	59	70	72	83
95–113	50	52	64	65	79	83	110

**Table 2:** The metrological parameters without filtering.

Selected Pairs of Two Signals (*x*_1_–*x*_2_), mm	Threshold *L*	Frequency Range *f*, kHz	Frequency Difference *c_ph_*, kHz	Mean Relative Error ${\bar{\delta }}_{c_{ph}}$, %
25–43	0.1	263–311	48	0.56
48–66	0.16	199–320	121	1.2
95–113	0.35	117–329	212	2.36

**Table 3:** The metrological parameters obtained with filtering.

Selected Pairs of Two Signals (*x*_1_–*x*_2_), mm	Frequency Range f,kHz	Frequency Difference $c_{ph},kHz$	Mean Relative Error ${\bar{\delta }}_{c_{ph}}$, %	Standard Deviation of the Measurement Model $\sigma_{{∆}_{mod}}$, m/s
25–43	164–424	260	0.1	1.3
48–66	165–418	253	0.12	1.7
95–113	167–405	238	0.13	1.3

**Table 4:** Determined filter bandwidths for selected central frequencies.

Distance Between the Signals (*x*_1_–*x*_2_), mm	Central Frequencies of the Filter, kHz					
	100	150	200	250	300	350
	Bandwidth of the Filter Δ*B*, kHz					
69–87	50	50	50	90	120	127
70–85	50	50	50	50	60	115
96–112	50	50	55	55	70	70
120–135	40	48	55	55	75	86
165–182	45	48	60	60	50	100

**Table 5:** The metrological parameters without filtering from experimental signals.

Selected Pairs of Two Signals (*x*_1_–*x*_2_), mm	Threshold *L*	Frequency Range $c_{ph}$, kHz	Reconstructed Frequency Difference $c_{ph}$, kHz	Mean Relative Error ${\bar{\delta }}_{c_{ph}},$ %	Standard Deviation of the Velocity (Dispersion Curve) $\sigma_{{∆}_{c_{ph}}}$, m/s
69–87	0.22	114–269	155	3.28	31.7
70–85	0.22	101–271	170	2.63	21.8
96–112	0.34	112–269	157	2.37	33.8
120–135	0.07	145–307	162	1.20	8.5
165–182	0.14	166–303	137	1.55	15.2

**Table 6:** The metrological parameters obtained with filtering from experimental signals.

Selected Pairs of Two Signals (*x*_1_–*x*_2_), mm	Frequency Range $c_{ph}$, kHz	Reconstructed Frequency Difference $c_{ph}$, kHz	Mean Relative Error ${\bar{\delta }}_{c_{ph}}$, %	Standard Deviation of the Velocity (Dispersion Curve) $\sigma_{{∆}_{c_{ph}}}$, m/s
69–87	105–323	218	1.50	10.5
70–85	105–335	230	1.33	12.3
96–112	104–341	237	0.41	2.00
120–135	107–336	229	0.40	6.2
165–182	106–333	227	0.26	1.3

**Table 7:** Summary of the uncertainty budget of the measurement of velocity for the *A*_0_ mode.

Sources of Combined Standard Uncertainty	Standard Uncertainty σ, m/s	Distribution	Sensitivity Coefficient *W*	Uncertainty Contribution σ·W, m/s
Mathematical model	1.4	Gaussian	1	1.4
Velocity (dispersion curve)	6.5	Gaussian	1	6.5
Fluctuations of the Lamb wave’s frequency *f*	7.8	Gaussian	1	9.8
Distance between two points	$5.8\times 10^{-5}$ m	Rectangular	$2\times 10^{-5}$ s	2.9
Combined standard uncertainty				*u_c_* = 0.6%

The mean relative error is 0.8%, and the expanded uncertainty: *U* = ±1.2%, *p* = 95%. The relative uncertainty is computed based on a phase velocity of 1999 m/s at a frequency of 300 kHz.

## Data Availability

The data presented in this study are available on request from the corresponding author.
